# A novel polyhedral oligomeric silsesquioxane-modified layered double hydroxide: preparation, characterization and properties

**DOI:** 10.3762/bjnano.9.284

**Published:** 2018-12-19

**Authors:** Xianwei Zhang, Zhongzhu Ma, Hong Fan, Carla Bittencourt, Jintao Wan, Philippe Dubois

**Affiliations:** 1 State Key Laboratory of Chemical Engineering, College of Chemical and Biological Engineering, Zhejiang University, Hangzhou 310027, China; 2 Center of Innovation and Research in Materials and Polymers (CIRMAP), University of Mons, Place du Parc 23, B-7000 Mons, Belgium; 3 School of Materials Science and Engineering, Shaanxi Normal University, Xi’an 710119, China

**Keywords:** flammability, layered double hydroxide, polyhedral oligomeric silsesquioxane, thermal stability

## Abstract

A novel layered double hydroxide modified by octa-substituted carboxy-terminated polyhedral oligomeric silsesquioxane was prepared via a one-step method and characterized by Fourier-transform infrared spectroscopy, X-ray photoelectron spectroscopy, X-ray diffraction, transmission electron microscopy, scanning electron microscopy, elemental analysis, thermogravimetric analysis, and microscale combustion calorimetry (MCC). Results showed that the silsesquioxane modified-LDH (OLDH) revealed an increase in the interlayer distance, nanoscale plate-like morphology of primary particles, and improved thermal stability. A synergistic effect between the siloxane moiety and Mg–Al hydroxide was found during thermal degradation, and confirmed by the study of degradation kinetics together with the analysis of the surface morphologies and elemental components of char residues. Moreover, in contrast to conventional organic modified LDH (e.g., dodecylbenzenesulfonate-LDH), the MCC results showed a significant decrease in the heat release rate and total heat release, indicating the low flammability of OLDH.

## Introduction

Layered double hydroxides (LDHs) are typical host–guest materials, with unique lamellar structures composed of positively charged brucite-like layers and an interlayer region containing charge compensating anions and solvate molecules. The metal cations are octahedrally surrounded by hydroxide ions, and the octahedra units share their edges connecting to form infinite 2D sheets [1]. [M^2+^_1−_*_x_*M^3+^*_x_*(OH)_2_]*^x^*^+^·A*^n^*^−^*_x_*_/_*_n_*·*y*H_2_O is the generic structural formula for LDHs, where M^2+^, M^3+^ and A*^n^*^−^ represent divalent metal cations (e.g., Mg^2+^, Zn^2+^, Ni^2+^, Co^2+^), trivalent metal cations (e.g., Al^3+^, Fe^3+^, Mn^3+^, Cr^3+^) and interlayer anions (e.g., NO_3_^−^, Cl^−^, CO_3_^2−^), respectively [2–3]. The stoichiometric coefficient *x* is typically in the range of 0.17–0.33, and the species of metallic ions and intercalated anions are variable, giving rise to versatile isostructural LDHs [1–4].

In recent years, LDHs have found wide application in polymer nanocomposites because they are highly tunable and can observably improve the flame retardancy of polymers [5–13]. Due to size effects, the strong hydrophilicity and the strong affinity between the hydroxide layers, LDHs are difficult to homogeneously and stably disperse in polymer matrices. Therefore organic modification of LDHs is essential before using them as nanofillers. So far, many organic modifiers have been successfully used to modify LDHs. One of the most commonly used is sodium dodecylbenzene sulfonate (SDBS) [7,14]. Other modifiers include a variety of sulfate and phosphate surfactants [15–17], aliphatic and aromatic mono- and dicarboxylic acids [18–19], amino acids [20], anionic dyes [11], and compounds of biological origin, such as β-cyclodextrin and eugenol derivatives [9–10,13,21]. However, most of these current modifiers do not have satisfactory thermal stability and are flammable, reducing the thermal and fire stability of the LDHs after modification, which is expected to eventually limit the potential for applications of LDHs in polymer composites.

Polyhedral oligomeric silsesquioxanes (POSS) are a class of well-defined nanostructured molecules, which contain a silica cage core and externally attached organic substituents [22]. These 3D nanoclusters follow the basic composition formula of R*_n_*(SiO_1.5_)*_n_* and have a size of 1 to 3 nm in diameter when the vertex groups (R) are included. The siloxane cage in POSS provides the thermally and chemically robust framework, while the organic functionality can be chemically modified in a controlled manner, making POSS a class of promising nanoscale building blocks for advanced functional nanomaterials [22–24]. Besides, the unique inorganic–organic architecture also endows POSS with low dielectric constant, high thermal and oxidation resistance [23,25]. And unlike traditional organic compounds, POSS chemicals are odorless and environmentally friendly as they release no volatile organic components. There are reports about the successful introduction of acid groups such as –COOH in POSS structures [26–27], making it possible to use POSS as ionic intercalators. However, there is scarcely any report on the use of POSS to intercalate LDHs.

**Figure 1:**
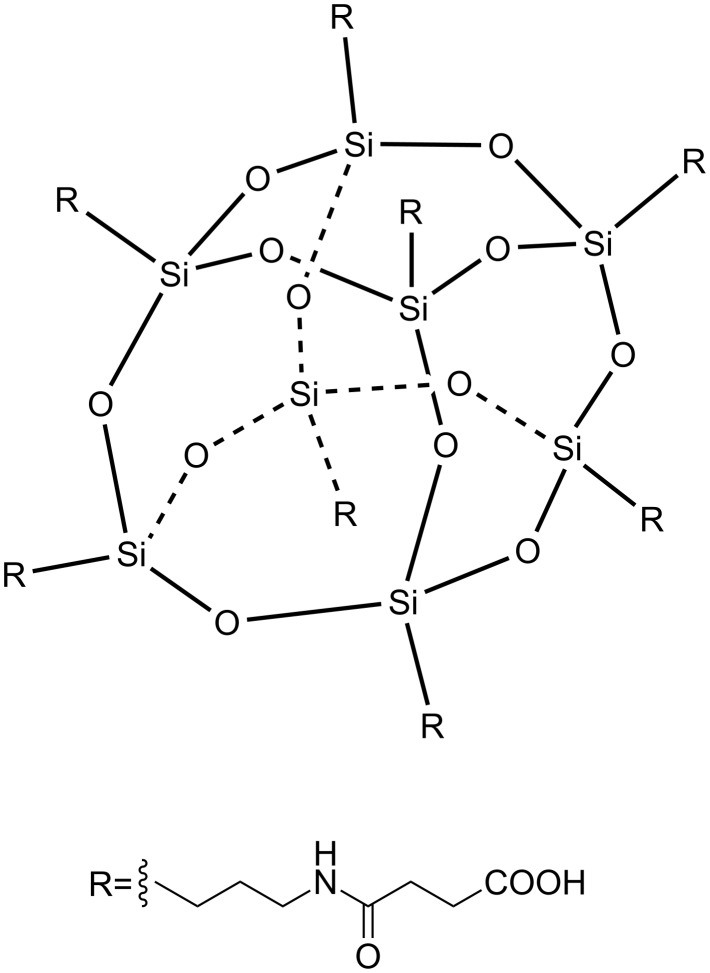
Chemical structure of OCPS (C_56_H_96_N_8_O_36_Si_8_).

Aiming to develop an organically modified LDH with satisfactory thermal stability and low flammability, an octa-substituted carboxy-terminated polyhedral oligomeric silsesquioxane (OCPS, Figure 1) was synthesized and originally employed to modify Mg–Al LDH in this work. Herein, OCPS was chosen as the candidate POSS intercalator because of the following advantages: (i) The long alkyl chains anchoring on the rigid POSS cage are flexible, reducing the resistance against the formation of layered structure. (ii) The OCPS molecule is rich in carboxylate groups, which could counterbalance multiple positive sheet charges. (iii) The high hydrocarbon content of the intercalator is helpful in improving the affinity of LDH to carbon-based materials. The OPCS-modified LDH (OLDH) was designed and prepared using a one-step route, followed by the systematical investigation of its structure, morphology, thermal stability and combustion behavior. It was expected that the incorporation of the POSS moiety into LDH structure would result in good maintenance of thermal stability and improved fire resistance than traditional organically modified LDHs, e.g., DBS-LDH.

## Results and Discussion

### Synthesis and structure characterization of OAPS and OCPS

**Scheme 1:**
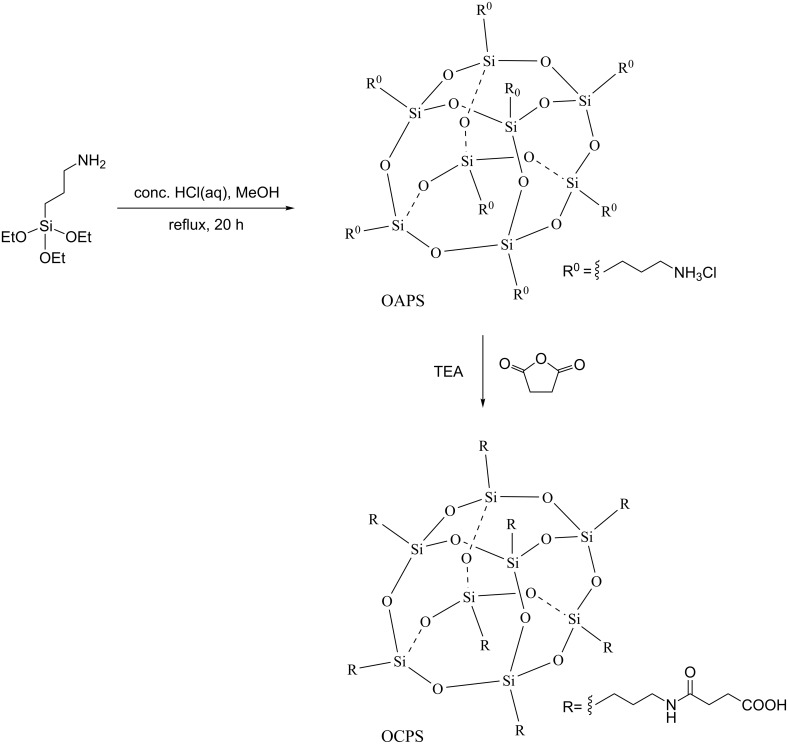
Synthesis of OCPS.

The route for the synthesis of octa-substituted carboxy-terminated POSS (OCPS) is depicted in Scheme 1. First, octa-ammonium POSS (OAPS) was synthesized employing a modified refluxing method [28–30]. Thereafter, the acylation reaction of OCPS with succinic anhydride was carried out to afford OCPS.

**Figure 2:**
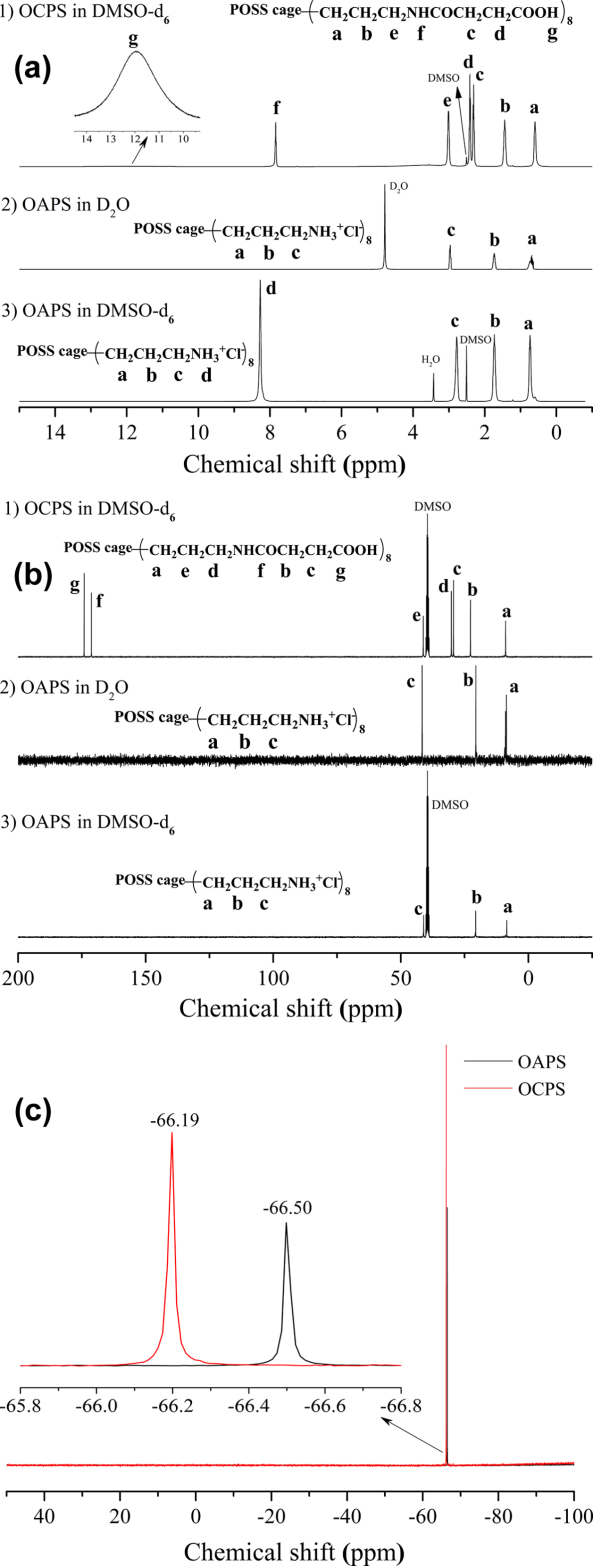
(a) ^1^H NMR, (b) ^13^C NMR and (c) ^29^Si NMR spectra of OAPS and OCPS.

**Figure 3:**
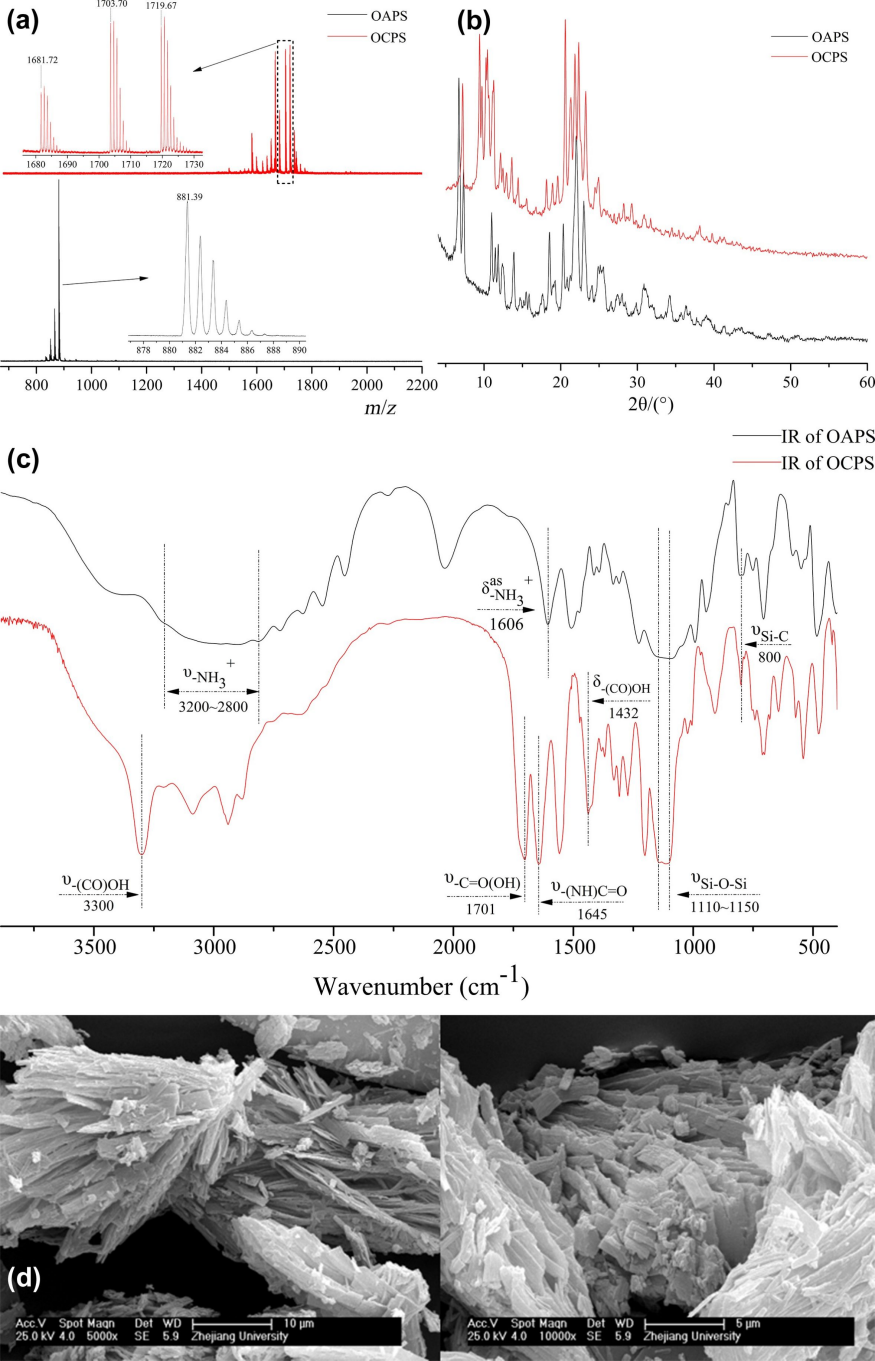
(a) MALDI-TOF-MS spectra, (b) XRD patterns and (c) FTIR spectra of OAPS and OCPS; (d) SEM images of OCPS.

The ^1^H NMR, ^13^C NMR and ^29^Si NMR spectra of OAPS and OCPS presented in Figure 2 and the MALDI-TOF-MS spectra, XRD patterns and FTIR spectra of OAPS and OCPS in Figure 3a–c reveal the well-defined structures. The SEM images of OCPS in Figure 3d show that OCPS has a micrometer-sized strip geometry. The details are as follows: (1) for OAPS: ^1^H NMR (DMSO-*d*_6_) δ 8.27 (s, N*H*_3_, 24H), 2.78 (s, C*H*_2_N, 16H), 1.73 (s, SiCH_2_C*H*_2_, 16H), 0.69 (m, SiC*H*_2_, 16H); ^1^H NMR (D_2_O) δ 2.96 (m, C*H*_2_N, 16H), 1.73 (m, SiCH_2_C*H*_2_, 16H), 0.70 (m, SiC*H*_2_, 16H); ^13^C NMR (DMSO-*d*_6_) δ 41.03 (s, *C*H_2_N), 20.64 (s, SiCH_2_*C*H_2_), 8.46 (s, Si*C*H_2_); ^13^C NMR (D_2_O) δ 41.63 (d, *C*H_2_N), 20.55 (d, SiCH_2_*C*H_2_), 8.76 (m, Si*C*H_2_); ^29^Si NMR (DMSO-*d*_6_) δ −66.50 (s); MALDI-TOF-MS (DHB matrix, *m*/*z*): [M + H − 8HCl]^+^ calcd 881.29; found, 881.39 (100%); FTIR (KBr): υ(–NH_3_^+^) 3200–2800 cm^−1^, δ_as_(–NH_3_^+^) 1606 cm^−1^, υ(Si–O–Si) 1175–1060 cm^−1^, υ(Si–C) 800 cm^−1^; XRD: 2θ 6.72°, 7.32°, 11.00°, 18.55°, 20.34°, 22.03°, 23.01°, 25.57°. (2) for OCPS: ^1^H NMR (DMSO-*d*_6_) δ 11.95 (s, br, COO*H*, 8H), 7.83 (t, *J* = 4.9 Hz, N*H*C=O, 8H), 3.00 (d, *J* = 5.3 Hz, C*H*_2_N, 16H), 2.41 (t, *J* = 6.6 Hz, C*H*_2_COOH, 16H), 2.30 (t, *J* = 6.5 Hz, C=OC*H*_2_, 16H), 1.43 (s, CH_2_C*H*_2_CH_2_, 16H), 0.59 (s, SiC*H*_2_, 16H); ^13^C NMR (DMSO-*d*_6_) δ 174.11 (s, *C*OOH), 171.24 (s, NH*C*O), 41.20 (s, CH_2_*C*H_2_CH_2_), 30.18 (s, *C*H_2_NH), 29.33 (s, *C*H_2_COOH), 22.67(s, C=O*C*H_2_), 8.92 (s, Si*C*H_2_); ^29^Si NMR (DMSO-*d*_6_) δ −66.19 (s); MALDI-TOF-MS (DHB matrix, *m*/*z*): [M + H]^+^ calcd 1681.42, found 1681.72; [M + Na]^+^ calcd 1703.70; found, 1704.70; [M + K]^+^ calcd 1719.37; found, 1719.67; FTIR (KBr): υ(-CO*OH*) 3300 cm^−1^, δ(–CO*OH*) 1432 cm^−1^, υ(–*CO*OH) 1701 cm^−1^, υ(–NH*C***=***O*) 1645 cm^−1^, υ(Si–O–Si) 1150–1110 cm^−1^, υ(Si–C) 800 cm^−1^; XRD: 2θ 7.23°, 9.41°, 9.75°, 10.25°, 10.43°, 11.30°, 20.61°, 21.30°, 21.85°, 22.33°, 23.25°.

### Synthesis and structure characterization of OCPS-modified Mg–Al LDH

**Scheme 2:**
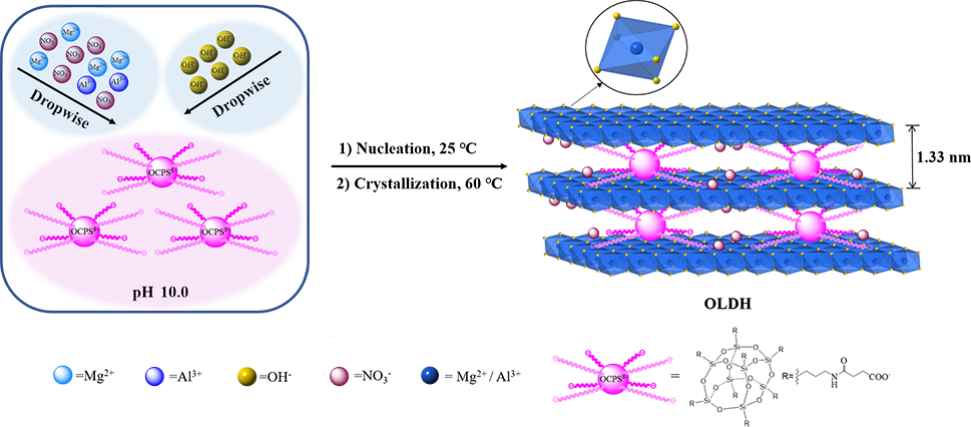
Schematic diagram of the synthesis of OLDH.

The OCPS-modified Mg–Al LDH (denoted OLDH) was prepared via a one-step route [14] (Scheme 2). Unmodified pristine NO_3_^−^-Mg–Al LDH (denoted NLDH) was prepared in the same way as a reference sample.

### FTIR and XPS

**Figure 4:**
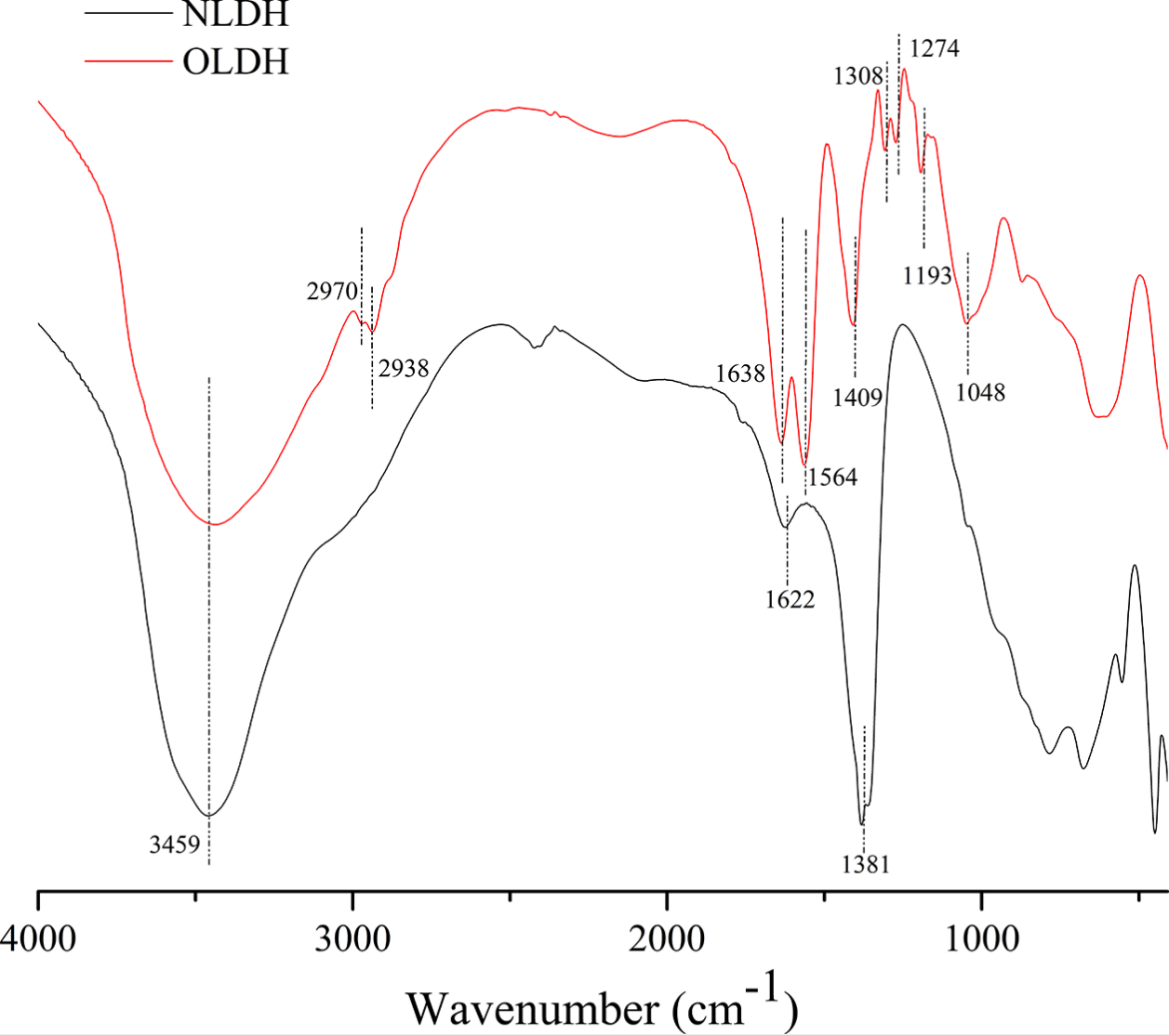
FTIR spectra of NLDH and OLDH.

FTIR is very useful in confirming the structures of LDH materials at the molecular level, as it provides important information about the interlayer anions. Figure 4 shows the FTIR spectra of NLDH and OLDH. The broad band in the range of 3700–3200 cm^−1^ is assigned to the O–H stretching of the metal hydroxide layer and interlayer water molecules. Another band characteristic of the interlayer water is shown in the broad and weak peak around 1622 cm^−1^ in spectrum of NLDH, which is covered by the strong absorption bands of carboxylic ions and amide groups in the case of OLDH. As for NLDH, the strong absorption around 1381 cm^−1^ confirms the presence of interlayer NO_3_^−^, and the bands recorded below 800 cm^−1^ originate from the vibration of metal–oxygen bonds and from lattice vibrations associated with metal hydroxide sheets [15]. The introduction of OCPS anions after modification is confirmed by the new characteristic vibration bands detected for –COO^−^ (C=O asymmetric stretching at 1564 cm^−1^ and symmetric stretching at 1409 cm^−1^), –NH–C=O (C=O stretching at 1638 cm^−1^, C–N stretching and N–H bending around 1310–1230 cm^−1^), alkyl (C–C–C asymmetric stretching at 1193 cm^−1^, C–H stretching at 3000–2800 cm^−1^), and Si–O–Si (asymmetric stretching around 1000–1200 cm^−1^). The disappearance of the strong stretching vibration for carboxylic acid C=O around 1701 cm^−1^ indicates that OCPS was almost totally converted into its ion state. Despite the possible fact that the band of residual nitrate might be mixed with the symmetric stretching of carboxylic ions around 1409 cm^−1^, the weakened absorption strength compared with that of NLDH suggests the nitrate was exchanged with OCPS carboxylic ions to a large extent. These results reveal the presence of OCPS anions in the OLDH structure and are consistent with the following XPS, XRD and elemental analysis results.

**Figure 5:**
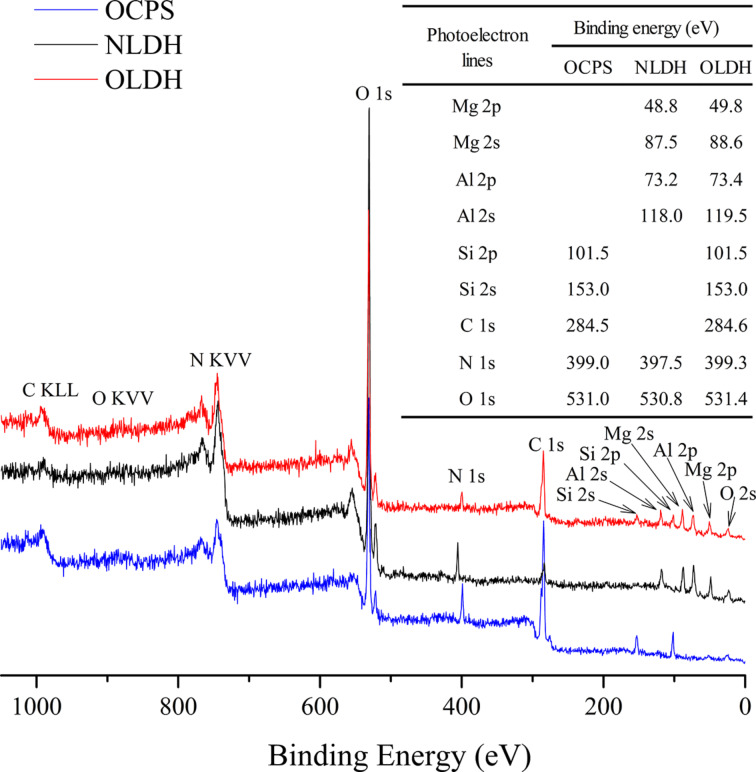
XPS spectra of OCPS, NLDH and OLDH.

The presence of the elements Mg, Al, Si, O, C and N in OLDH is further confirmed by XPS. The survey spectra illustrated in Figure 5 show the peaks of electrons emitted from the Mg 2p, Mg 2s, Al 2p, and Al 2s core levels. Compared to NLDH, these peaks in the brucite-like lattice show a small shift toward higher binding energies, probably arising from the interferences of OCPS intercalators. A similar phenomenon has been reported before in the case of carbon black nanoparticles modified Ni–Fe LDH [31].

### XRD

**Figure 6:**
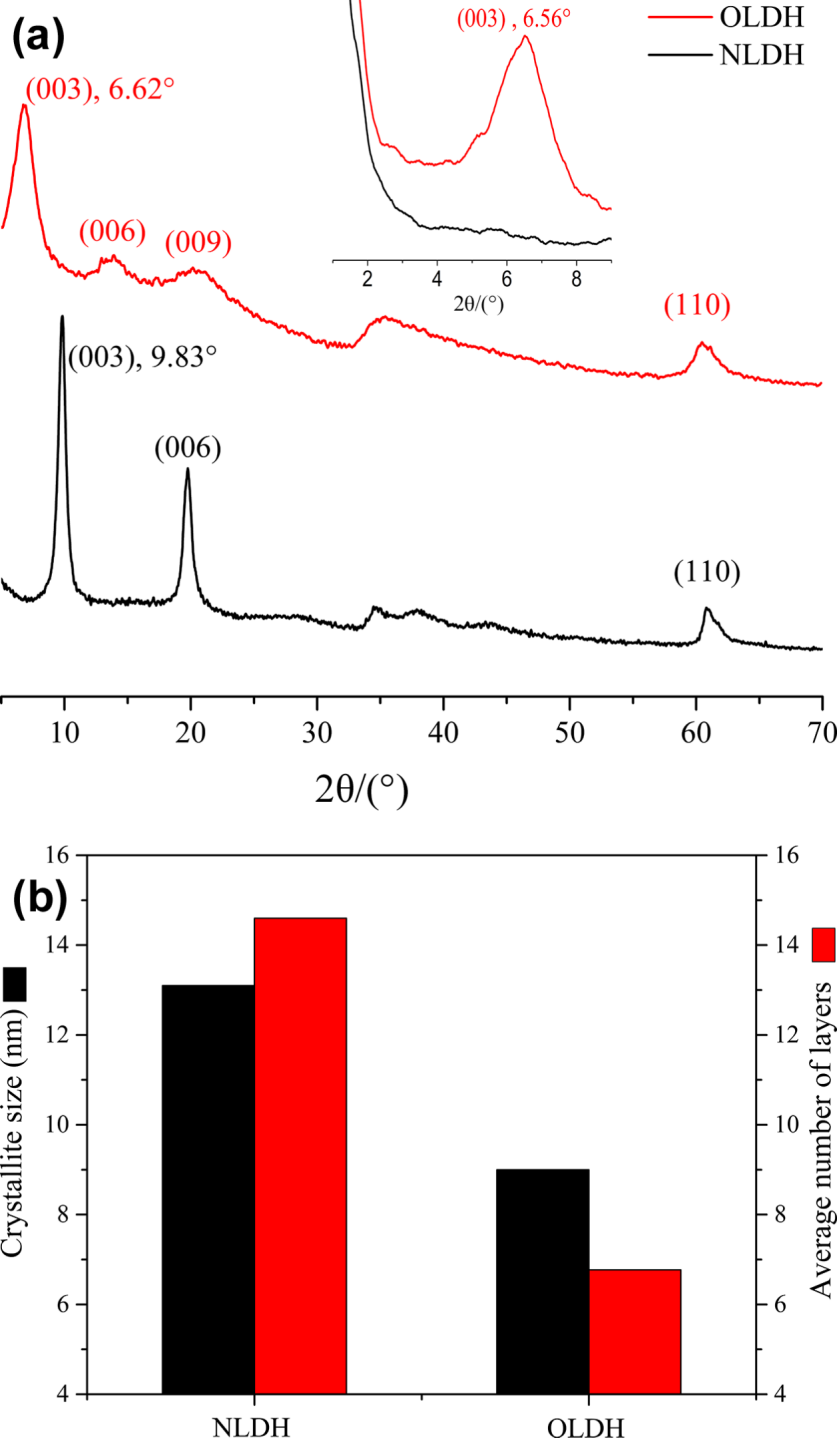
(a) XRD patterns (WAXS: 5–70°, SAXS: 1–10°) and (b) crystallite size (thickness in the normal direction to the (00*l*) planes) of NLDH and OLDH.

The XRD patterns of NLDH and OLDH are presented in Figure 6a. These patterns exhibit a series of well-defined (00*l*) Bragg reflections with the basal spacing. In addition, higher-order reflections appear at low angles, and there are in-plane (110) peaks at high angles. The measurements indicate the formation of a layer-by-layer ordered stacking structure of Mg–Al LDH crystallites. The first basal reflection (003) of NLDH at 2θ = 9.83° corresponds to an interlayer distance of 0.90 nm. After the modification with OCPS, the position of the (003) reflection shifts to 6.62°, indicating an interlayer distance of 1.33 nm of OLDH. The enlargement of the interlayer spacing could be attributed to the relatively large intercalator molecules, and the decrease in peak pattern sharpness suggests a decline in crystallinity. The absence of any distinguishable reflection of OCPS or NLDH in the OLDH pattern indicates that unbonded excess OCPS was totally removed and no unmodified LDH phase is formed. This shows that OCPS anions can be efficiently intercalated within the LDH layers using the one-step method. The crystallite size and the average number of layers estimated from crystallite size and interlayer distance are plotted in Figure 6b. Compared to unmodified LDH, OLDH has a smaller crystallite size and shows a nearly two-fold reduction of the average number of layers. This drastic reduction could be explained as follows: With the interlayer spacing being increased by OCPS molecules, a large-size stacking of LDH laminae is sterically hindered [8].

**Figure 7:**
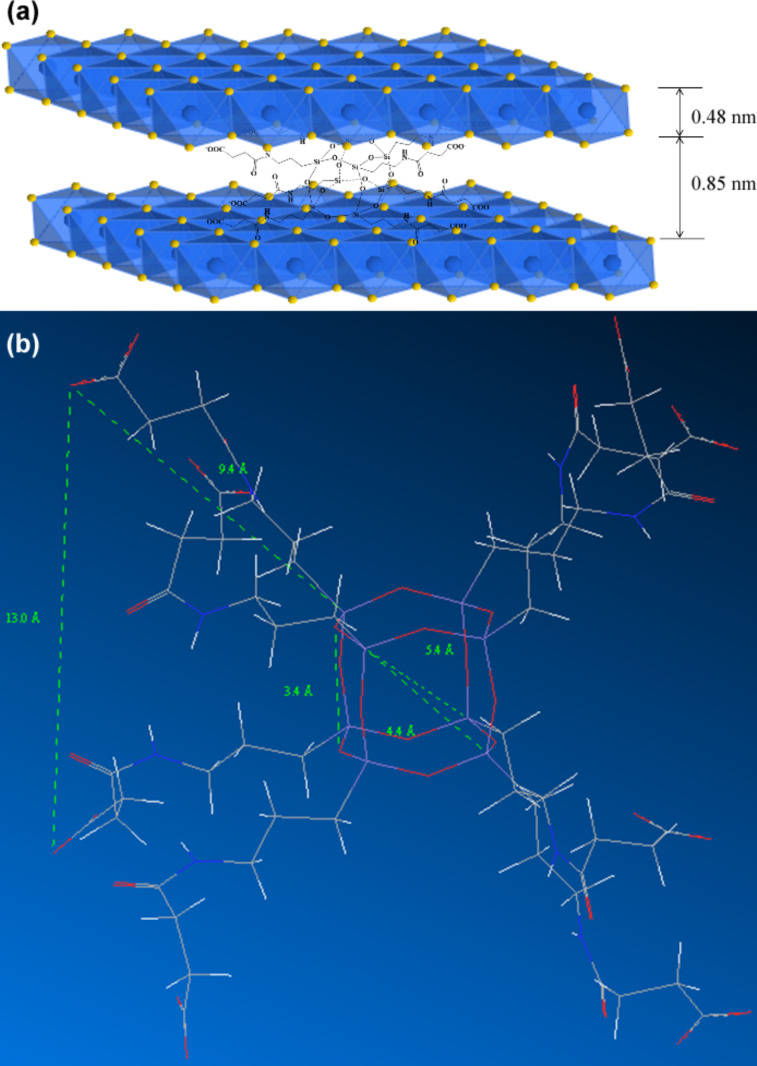
(a) Possible crystallographic structure of OLDH; (b) Energy-minimized molecule structure of an OCPS anion (geometrically optimized by chem3D and HyperChem).

Figure 7a schematically shows the possible brucite-like crystallographic structure of OLDH. The thickness of Mg–Al hydroxide sheets is about 0.48 nm [32]. Here the intergallery spacing in the Mg–Al hydroxide sheets for NLDH and OLDH were calculated as 0.42 and 0.85 nm, respectively. It is a clear advantage of the one-step method that the possible presence of small organic anions in the interlayer region along with intercalator of much larger size hardly affects the interlayer distance [14]. The intergallery spacing of OLDH crystallites is mainly controlled by OCPS anions despite the possible existence of co-intercalating NO_3_^−^ ions. Figure 7b shows the energy-minimized molecule structure of the OCPS anion, with a dimension of the POSS core in the range of 0.34–0.54 nm and an approximate length of each POSS arm of 0.94 nm. The side length of the whole cubic-like molecule is calculated as ca. 1.30 nm, larger than the resulting intergallery spacing of OLDH, suggesting that OCPS anions in OLDH interlayers are probably in a restricted state. One possible explanation for this could be that the linear linkages present in the POSS structure are relatively flexible compared to the rigid silica cage, and that these long arms are rearranged under the action of forces arising during the synthetic process and from interlayer surroundings.

Furthermore, the interlayer features of OLDH are compared to those of traditional LDHs synthesized in a similar way, and the ratios between intergallery spacing and size of the intercalator molecules (*H*/*L*) are summarized in Table 1. For LDHs intercalated with small inorganic molecules like NO_3_^−^ and CO_3_^2−^, the ration *H*/*L* reaches values as high as 3–4, and the values are usually in the range of 1.1–1.8 in the case of LDHs modified with linear organic anions. In contrast, the LDH modified with OCPS seems to have a denser interlayer packing. Previous literature suggests that the intercalation with linear organic molecules, e.g., surfactants, into pristine LDH can significantly weaken the hydrogen-bonding interactions between interlayer water, interlayer anions and the metal-hydroxide sheets. This largely reduces the contribution of the water layer accommodated in the interlayer region to the basal spacing [15,19,33]. Here, it is proposed that the less strongly bound interlayer water, which is further verified later by TGA results, might be another important factor accounting for the “compact structure” of OLDH.

**Table 1:** Ratio between intergallery spacing and intercalator molecule size (*H*/*L*) of LDHs synthesized via the one-step route with Mg/Al (mol) = 2 [9,11,14].

types of intercalators		*H*/*L*

inorganic small molecules	NO_3_^−^, CO_3_^2−^	3–4
linear molecules	SDBS	1.16
	acid yellow 36^a^	1.56
	acid red 88^b^	1.78
	SIEPDP^c^	1.22

^a^Sodium 3-(*p*-anilinophenylazo)benzenesulfonate; ^b^sodium 4(-2-hydroxy-1-naphthylazo)naphthalenesulphonate; ^c^((1,1,3,3-tetramethyldisiloxane-1,3-diyl)bis(propane-3,1-diyl))bis(2-methoxy-4,1-phenylene)bis(phenylphosphonochloridate).

### TEM and SEM

**Figure 8:**
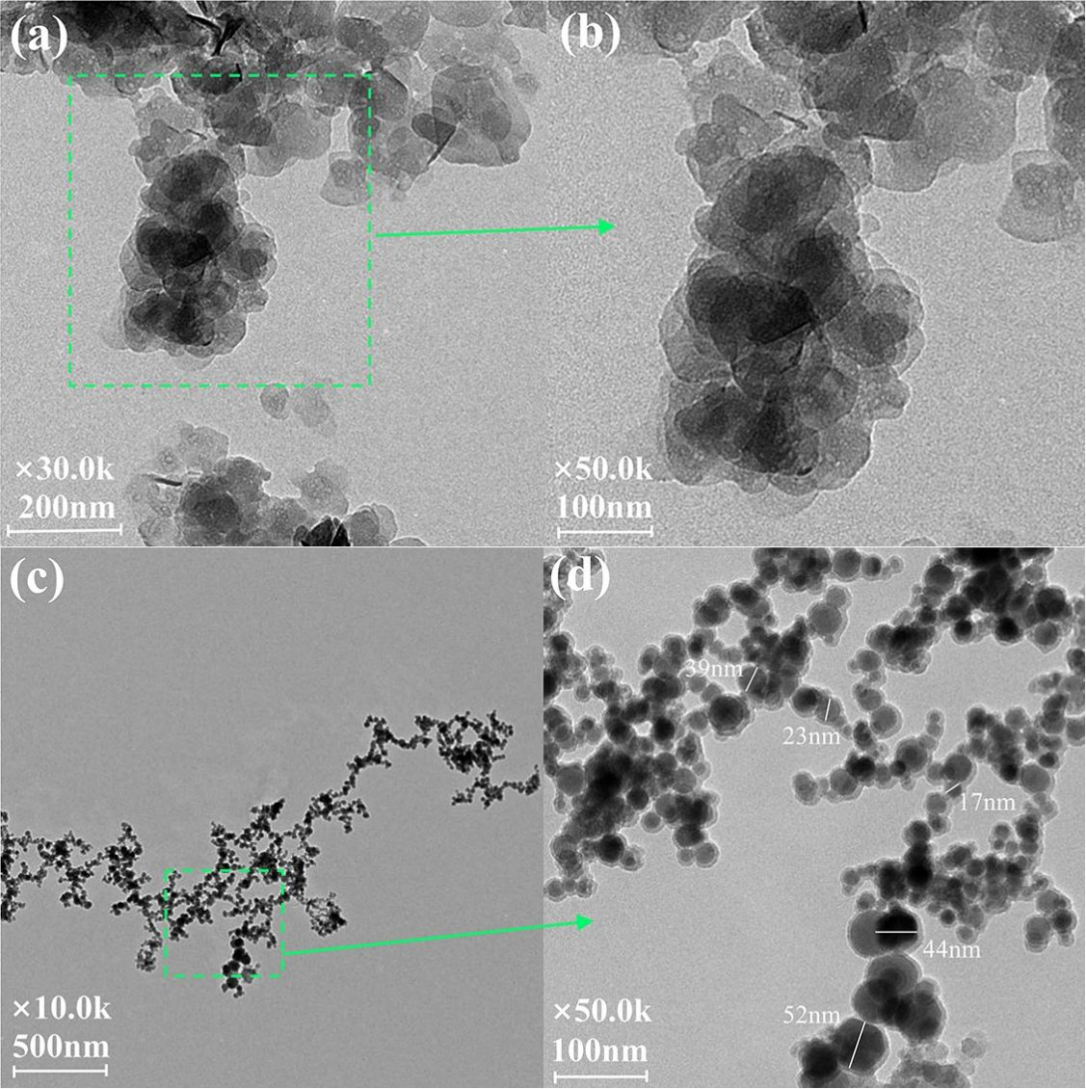
TEM images of (a, b) NLDH and (c, d) OLDH.

**Figure 9:**
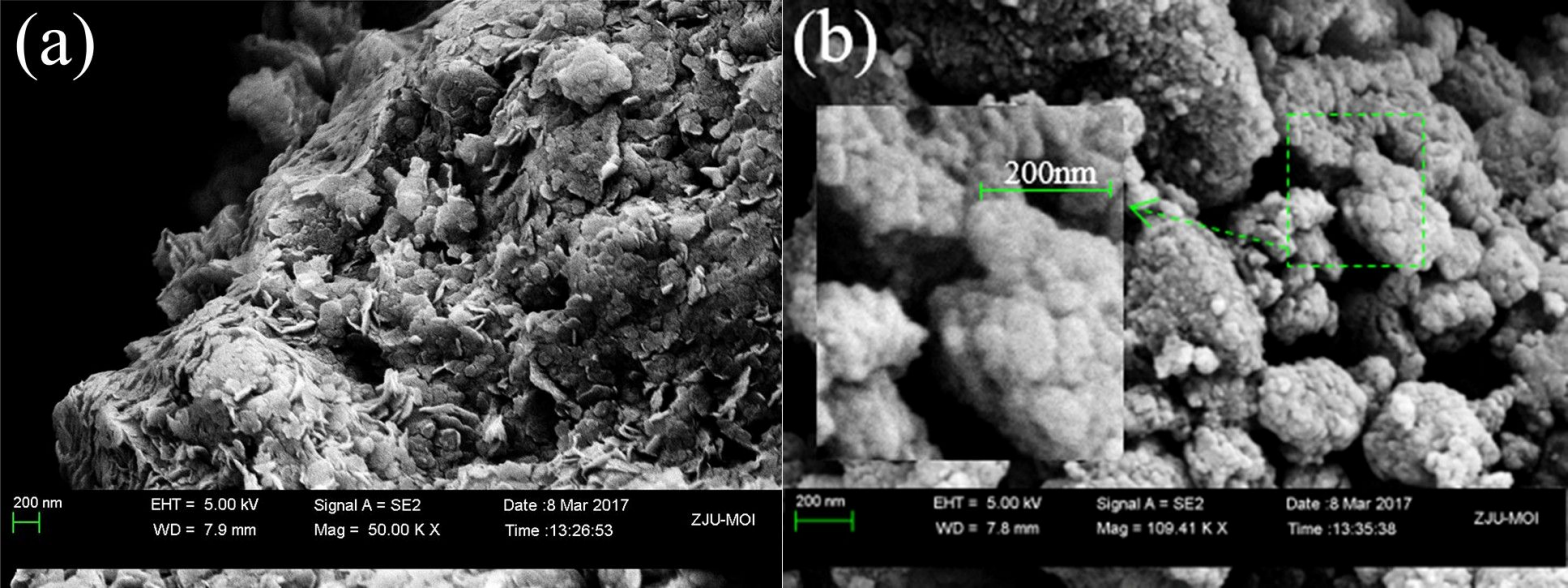
SEM images of (a) NLDH and (b) OLDH.

The morphology of the LDHs was investigated through TEM and SEM, and the images are shown in Figure 8 and Figure 9, respectively. As can be observed from the contrast in Figure 8a–b, the primary particles of NLDH exhibit plate-like geometries with sizes mainly ranging from 100 to 200 nm in diameter and without any defined outer shape. The irregular and arc-like edges of most particles might be an indication of the incomplete process of crystal growth. On the other hand, the primary particles of OLDH in Figure 7c–d are also found to be plate-like, with smaller sizes in the range of 10–50 nm in diameter. The intercalation of POSS anions does not significantly change the two-dimensional morphology of the LDH. The highly non-uniform sizes and imperfect edges of these particles are an implication for the incomplete growth of OLDH crystals, which might be ascribed to the huge steric hindrance of OCPS. Through sufficient and suitable post-synthesis treatment, a well-defined geometry of all particles might be obtained.

The SEM micrographs in Figure 9 show that both LDHs have an agglomerated morphology. The plate-like geometry and the dimensions of NLDH are roughly in accordance with the results obtained from TEM. However, as the thickness of the platinum coating layer is about 15 nm, close to the size of OLDH primary particles, the surface morphology of OLDH is not easily verified and seems to remind of stacked nuggets.

### Elemental analysis

The OCPS-modified LDH can be represented by the chemical formula Mg_2_Al(OH)_6_OCPS*_x_*_/8_(NO_3_)_1−_*_x_*·0.4H_2_O, where OCPS is in its ionic state and *x* is the degree of intercalation of OCPS. Based on elemental analysis data of carbon [C (wt %)], *x* can be calculated from the following equation:


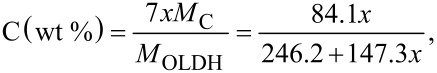


where *M*_C_ and *M*_OLDH_ are the molecular weights of carbon and OLDH, respectively. A calculation based on the results in Table 2 shows that OLDH has an intercalation degree of 50.8%, and the other anions remain NO_3_^−^ as the co-intercalator with a molar ratio of OCPS^8−^/NO_3_^−^ ≈ 1:8.

**Table 2:** Elemental analysis results of OLDH.

N (wt %)	2.34
C (wt %)	13.30
H (wt %)	5.00

### Thermal stability and combustion behavior

#### Thermal stability

**Figure 10:**
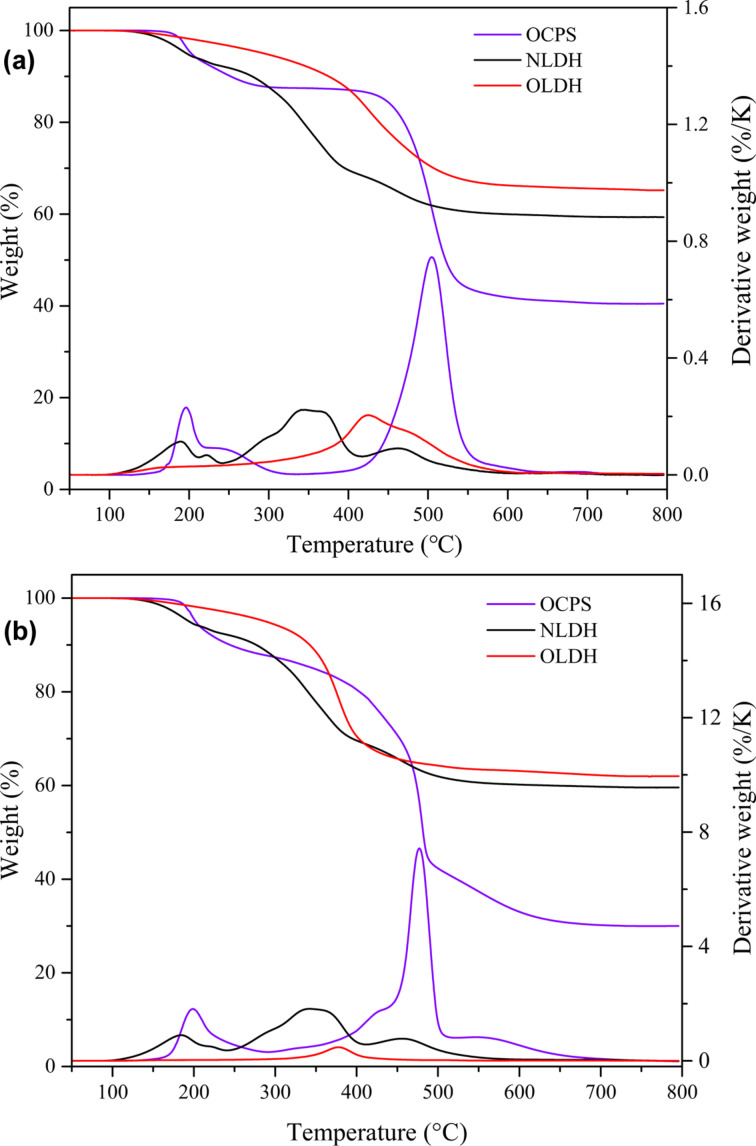
TG and DTG curves in (a) N_2_ and (b) air.

The thermal decomposition behavior was investigated to evaluate the thermal stability of LDHs, and the TGA thermograms are presented in Figure 10, with the measured data being summarized in Table 3. As can be seen in Figure 10a, the decomposition of NLDH under N_2_ atmosphere mainly undergoes three distinct steps, corresponding to the desorption process of interlayer water (up to about 240 °C), the partial and complete loss of OH^−^ from the hydroxide layer (245–345 °C and 345–420 °C) and the denitration of interlayer nitrate ions (420–580 °C) [3–4,34]. The curve of OLDH is much different and demonstrates a single major stage of weight loss, which is largely due to the release of gaseous products from the decomposition of OCPS^8−^ accompanied by the dehydroxylation of the host material. OLDH exhibits a significantly higher initial decomposition temperature (*T*_5%_, 97 °C higher), an enhanced temperature at the maximum rate of degradation (*T*_max_, 83 °C higher) and an improved residue yield at 800 °C (10% higher). Interestingly, as the initial decomposition temperature and char residue of OLDH are much higher than those of OCPS as well, some kind of synergistic effect might occur during the degradation process in the presence of POSS. Similar tendencies are also observed when the decomposition proceeds under air atmosphere (see Figure 10b) with the improvement being slightly decreased, owing to the more thorough decomposition of organic components. These observations clearly demonstrate that the thermal stability of OLDH is well-maintained and even enhanced in comparison to NLDH, and obviously surpasses most organically modified LDHs reported in the literature [9,12,14–15,21,35], as can be seen from the comparison of TG data listed in Table 3.

**Table 3:** Comparison of TG and DTG data between OCPS, NLDH, OLDH and other organically modified Mg–Al LDHs in N_2_ and air.

	*T*_5%_ (°C)		*T*_max_ (°C)		char yield (wt %, 800 °C)	
	N_2_	air	N_2_	air	N_2_	air

OCPS	203	202	196 505	199 477	40.5	30.0
NLDH	197	195	189 340–347 457	184 341–344 456	59.3	59.6
OLDH	294	289	423	378	65.2	62.0
DBS-LDH [14–15]	<120	<120	—	—	ca. 42	≤42
laurate-LDH [15]	—	<120	—	—	—	≤ 42
phytic acid-LDH [12]	233	—	340	—	51.5	—
DB-CD-BS-LDH [21]^a^	263–267	—	284–306 395–420	—	46.3–47.8	—
SIEPDP-LDH [9]^b^	ca. 200	—	—	—	49.1	—
sCD-SDBS-Ph-LDH [12]^c^	223–224	—	462–467	—	43.4–49.8	—
aminobenzoate-LDH [35]	<120	—	113, 350, 472	—	<40	—

^a^LDH modified with a β-cyclodextrin derivative functionalized with a controllable carbon–carbon double bond; ^b^LDH modified with a eugenol derivative containing silicon and phosphorus; ^c^LDH modified with hydroxypropyl-sulfobutyl-β-cyclodextrin (sCD), phytic acid (Ph) and sodium dodecylbenzene sulfonate (SDBS).

**Figure 11:**
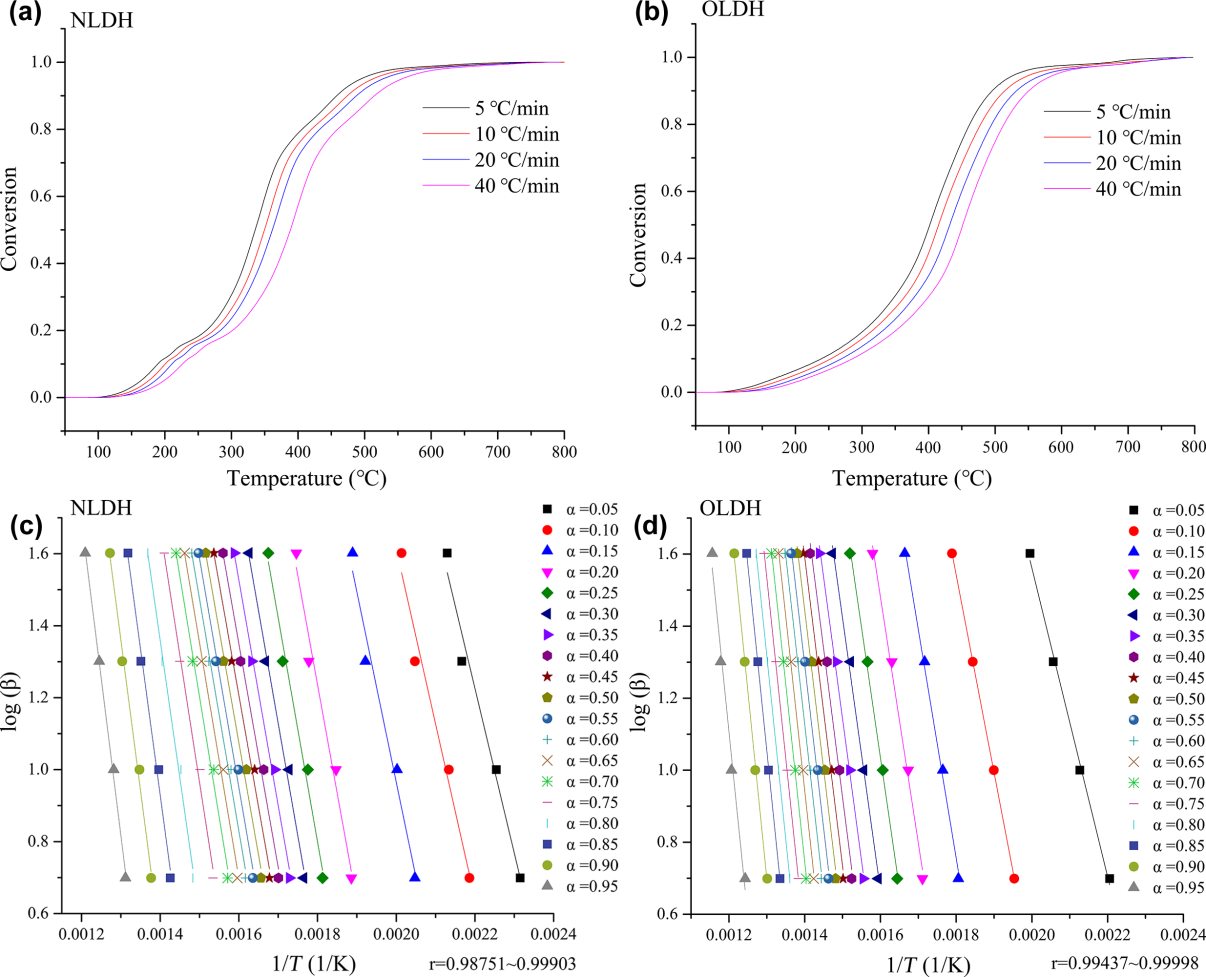
(a, b) Typical conversion curves at different heating rates and (c, d) plots of log(β) as a function of 1/*T* at different values of α.

To better understand the thermal degradation behavior, the kinetics of LDH degradation in a nitrogen atmosphere was studied using the Ozawa–Flynn–Wall (OFW) method [36–37]. The conversion (α, defined as the ratio of actual weight loss to total weight loss) as a function of the temperature at various heating rates (β) is shown in Figure 11a–b and the plots of log(β) versus 1/*T* for different values of α are presented in Figure 11c–d with decent linearity for both LDHs, indicating the applicability of the OFW method to our system in the investigated conversion range. The apparent activation energies (*E*α) are calculated from the slopes of these lines using the following expression:


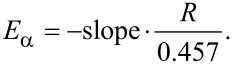


The fitting results and Eα data are summarized in Table 4. Results show that the apparent activation energy increases as a whole with increasing conversion. Combined with the TGA data and the data of α as a function of *T*, the *E*_α_ values of NLDH can be roughly divided into four distinct intervals, namely 80–90 kJ·mol^−1^ (desorption of interlayer water at α < 0.12), 113–114 kJ·mol^−1^ (primary dehydroxylation of the hydroxide layer at α = 0.22–0.50), 114–140 kJ·mol^−1^ (complete dehydroxylation at α = 0.50–0.80), 140–160 kJ·mol^−1^ (denitration at α > 0.85). On the other hand, the *E*_α_ values of OLDH increase continuously as α increases from 0.05 to 0.80 and remain in the range of 185–190 kJ·mol^−1^. It is obvious that the incorporation of the POSS structure into LDH can improve the activation energies over a wide range of reaction extents (α = 0.1–0.95), leading to the enhancement of thermal stability.

**Table 4:** Apparent activation energies *E*_α_ of LDH degradation in N_2_.

α	*E*_α_/kJ·mol^−1^	
	NLDH	OLDH

0.05	83.26	77.90
0.10	88.43	99.85
0.15	96.09	115.49
0.20	109.92	124.20
0.25	113.22	131.66
0.30	113.93	137.34
0.35	113.95	142.90
0.40	113.05	150.17
0.45	112.91	156.04
0.50	113.48	160.72
0.55	115.76	165.22
0.60	117.29	170.22
0.65	119.00	175.68
0.70	121.73	179.16
0.75	128.55	182.27
0.80	139.18	185.26
0.85	147.59	187.04
0.90	152.47	189.39
0.95	159.13	187.46

**Figure 12:**
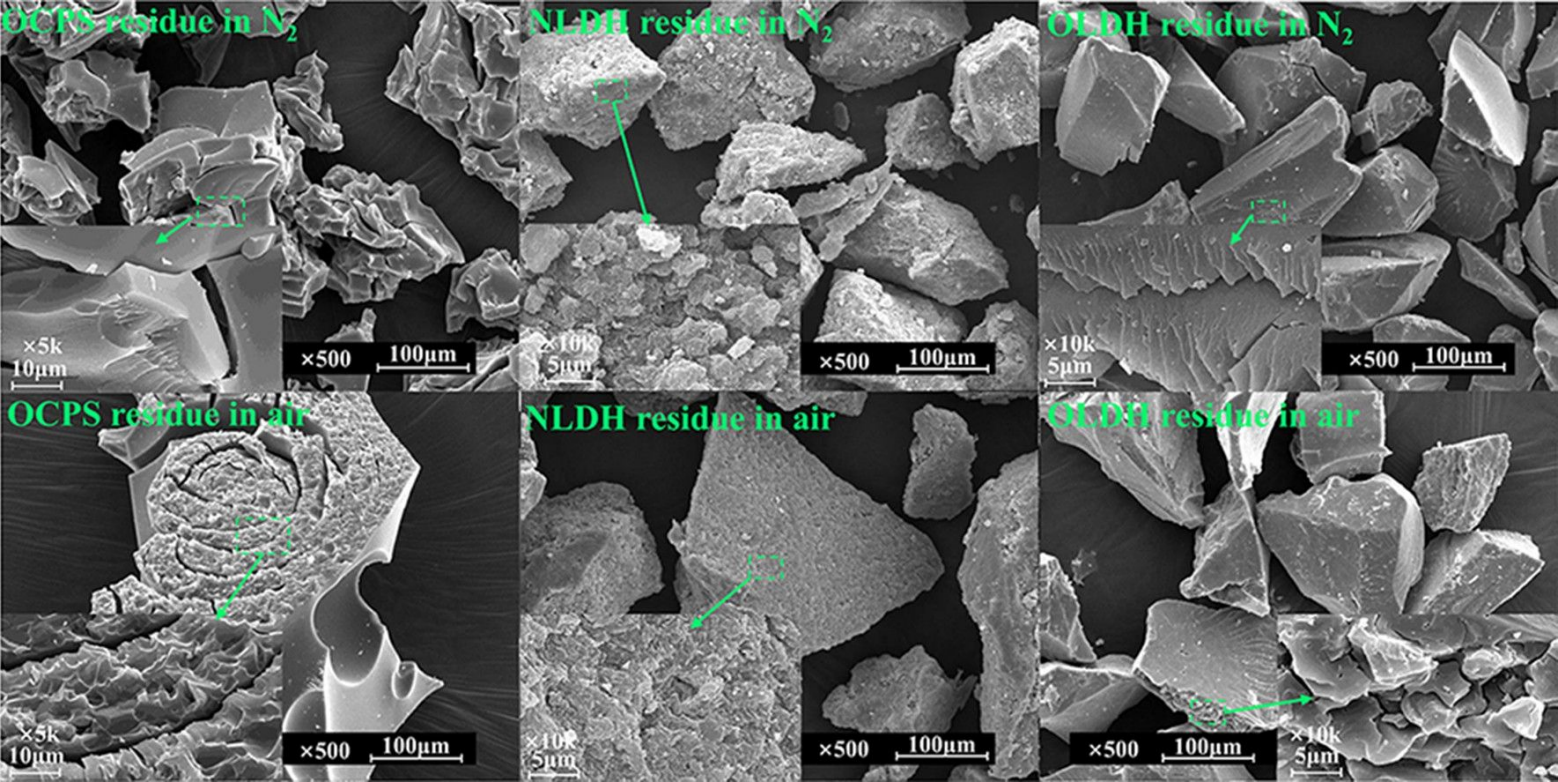
SEM morphology of char residues at 800 °C.

**Figure 13:**
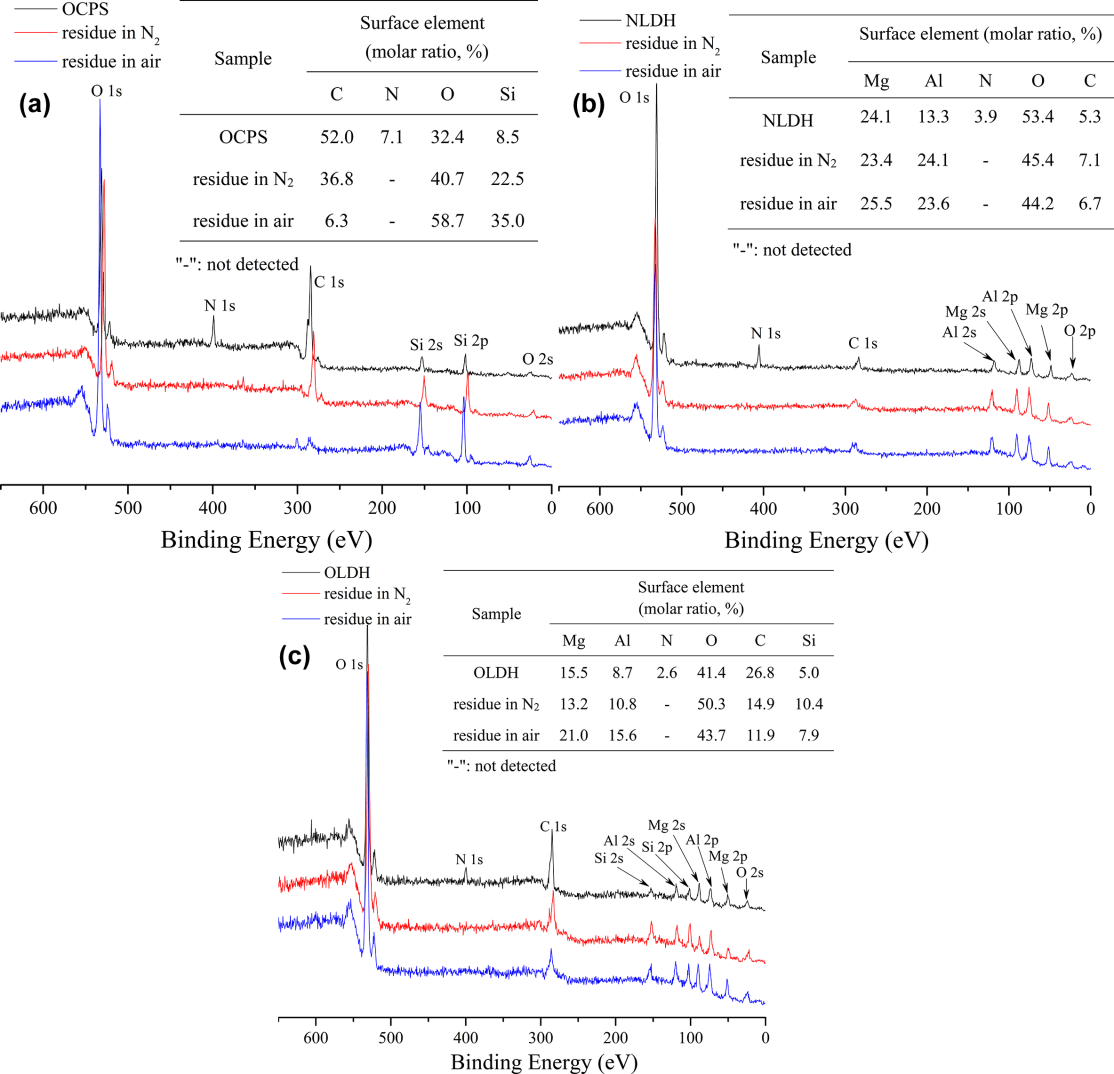
XPS measurements of surface components before and after thermal degradation of (a) OCPS, (b) NLDH and (c) OLDH).

Furthermore, the morphology of char residues after thermal degradation in both N_2_ and air was investigated through SEM with the images shown in Figure 12, and the change of surface components before and after degradation were examined through XPS with plots presented in Figure 13. The char of the intercalator OCPS exhibits a shrinkage with a very smooth and continuous dense surface in both N_2_ and air, due to the remarkable surface enrichment effect of silica during heating, which can be proved in the XPS spectra shown in Figure 13a. In the case of NLDH, the structure of LDH layers was completely destroyed by dehydroxylation, resulting in the formation of random stacking of mixed oxides containing Mg and Al [34]. The surface after degradation in air seems not as rough and coarse as that after degradation in N_2_, which might be caused by the different gas release process at high temperatures. The situation regarding OLDH is found to be very unique. The surfaces are much smoother and more compact compared to NLDH, demonstrating the char features of silicone materials. In more detail, the char of OLDH after degradation in N_2_ is composed of distinct laminas stacked together compactly and the char after degradation in air consists of dense blocks. It is proposed here that the flexible nature of polysiloxane and its weak intermolecular forces give rise to low surface energy and a high spreading coefficient. When heated, the siloxane moiety migrates to the surface and reacts with the metal oxides to form a continuous and compact condensed phase. This mixed carbonized protecting layer provides a good barrier to the transfer of heat and mass, preventing the inner layer against further decomposition and accelerating the formation of char. Evidence in favor of this assumption can be observed from the XPS results presented in Figure 13c. In addition, the significantly increased carbon content after degradation in air compared to that of OCPS also implies a synergistic effect of Mg–Al oxide and siloxane.

#### Combustion behavior

**Figure 14:**
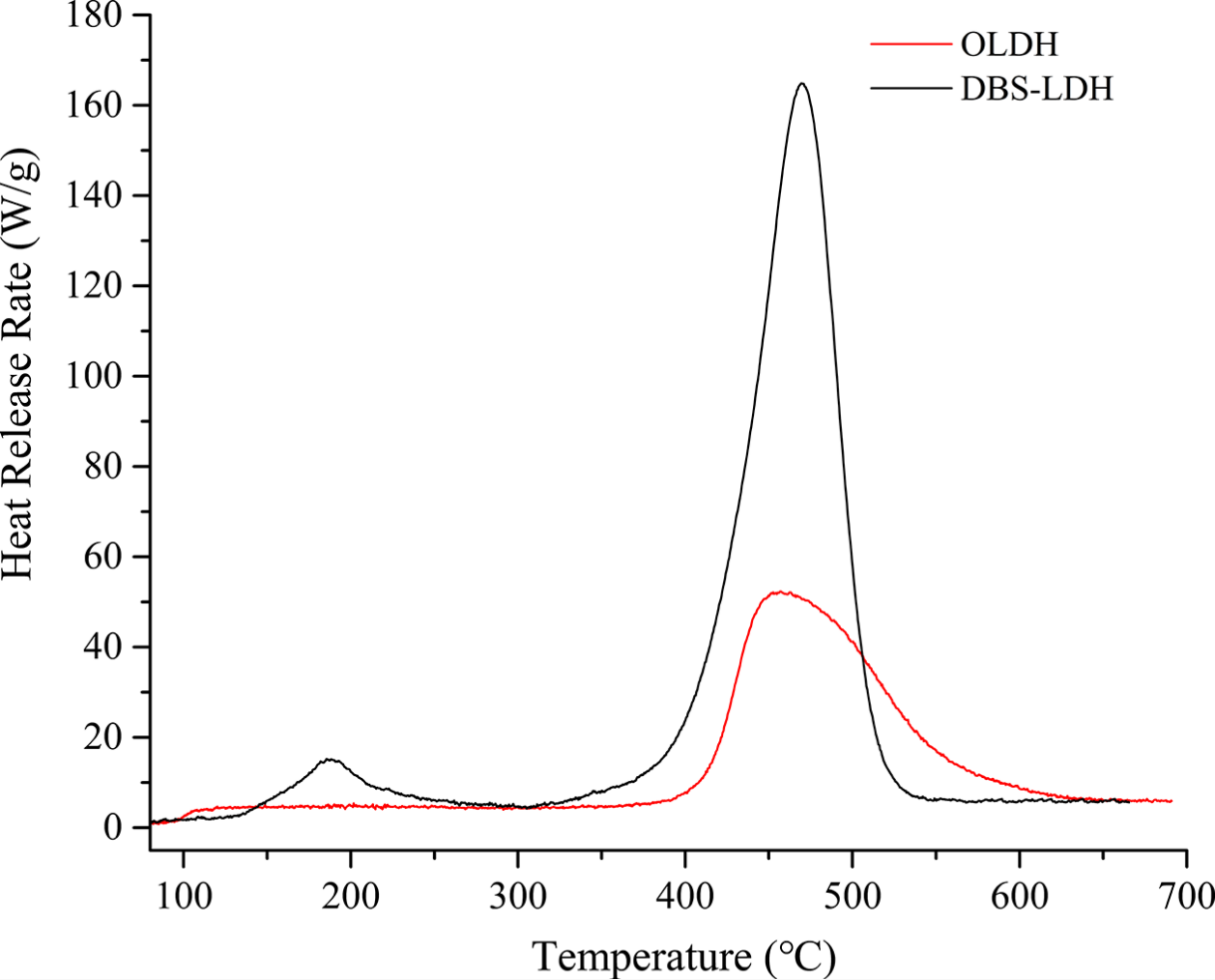
HRR curves of DBS-LDH adapted from [21] and OLDH.

The flammability of OLDH was assessed measuring the heat-release properties by using microscale combustion calorimetry (MCC). Heat-release rate (HRR), peak heat-release rate (pHRR), the heat-release capacity (HRC) and total heat release (THR) were measured. The combustion results were compared with that of the commonly used LDH modified with sodium dodecyl benzenesulfonate (SDBS, denoted as DBS-LDH). The HRR curves are plotted in Figure 14, and the corresponding combustion data are listed in Table 5. Typically, in the HRR curve of DBS-LDH, two stages of the combustion process can be distinguished by two separate peaks, which correspond to the combustion of a small amount of unbounded residual SDBS at lower temperature (around 186 °C) and the thermal decomposition of intercalated SDBS segments at higher temperature (around 467 °C). The excess SDBS is believed hard to be completely removed by simple purification procedures such as washing with water or centrifugation during LDH preparation [21]. In contrast, the HRR curve of OLDH displays a single peak around 458 °C, corresponding to the thermal decomposition of OCPS anions in LDH interlayers. The absence of a peak at lower temperatures suggests the purity of OLDH, which was consistent with the preceding IR and XRD results and also revealed the ease of purification of OLDH. Additionally, the temperature at the maximal pyrolysis rate (*T*_max_) showed no significant difference between these two LDHs. The pHRR and HRC values for OLDH were only 31.5% and 36.1%, respectively, of those for DBS-LDH, and the THR value was reduced by half. These results indicate that the potential hazards posed from a fire for OCPS-modified LDH are markedly decreased compared to SDBS-modified LDH.

**Table 5:** Combustion data measured from MCC.

	DBS-LDH	OLDH

pHRR (W·g^−1^)	165.0	52.0
HRC (J·g^−1^·K^−1^)	158.0	57.0
THR (kJ·g^−1^)	10.0	4.8
*T*_max_ (°C)	467	458

## Conclusion

In summary, a novel LDH modified with an octa-substituted carboxy-terminated POSS (OLDH) was designed and prepared via a one-step route. The structure and micromorphology of OLDH were confirmed by FTIR, XPS, XRD, TEM, SEM and elemental analysis. Results showed that OLDH exhibited an increased interlayer distance of 1.33 nm, a nearly two-fold reduction in the average number of layers compared to unmodified NO_3_^−^-LDH (NLDH), plate-like primary particles with diameters ranging from 10 to 50 nm, and an intercalation degree of about 50.8%. TGA investigation demonstrated the thermal stability of OLDH was effectively enhanced after the incorporation of the POSS structure, and the thermal degradation kinetics in N_2_ revealed that the apparent activation energies were impressively raised over a wide range of reaction extents (α = 0.1–0.95). Besides, a synergistic effect between the siloxane moiety and the Mg–Al hydroxide was found during thermal degradation, facilitating the char formation. Finally, the MCC results showed the combustion performance of OLDH far surpassed that of the commonly used SDBS-modified LDH.

This research offers a new approach to preparing modified LDHs with enhanced thermal stability and low flammability, and the POSS-modified LDH is expected to represent a powerful candidate for applications in LDH-based materials.

## Experimental

### Materials

All chemicals were of analytical grade and used as received without further purification unless otherwise noted. (3-Aminopropyl)triethoxysilane and succinic anhydride were purchased from Aladdin Co. Ltd (Shanghai, China). Methanol, chloroform, formic acid, magnesium(II) nitrate hexahydrate (Mg(NO_3_)_2_·6H_2_O), aluminum(III) nitrate nonahydrate (Al(NO_3_)_3_·9H_2_O), sodium hydroxide (NaOH), tetrahydrofuran (THF) and concentrated hydrochloric acid (36.0–38.0 wt %) were obtained from Sinopharm Chemical Reagent Co., Ltd (Shanghai, China). Ultrapure water with a resistance of 18.25 MΩ·cm was used for all experiments.

### Synthesis of octa-ammonium POSS and octa-substituted carboxy-terminated POSS

Octa-ammonium POSS (OAPS) was prepared using a modified refluxing method [28–30]. Typically, to a 1 L flask equipped with a condenser and a magnetic stirrer, (3-aminopropyl)triethoxysilane (30 mL), methanol (720 mL) and concentrated hydrochloric acid (60 mL) were added. The system was refluxed for 20 h under vigorous stirring and then cooled down to room temperature. The white precipitate was collected after precipitation in cold THF, filtration, and washing with cold methanol. The residue was then recrystallized in methanol, filtrated, and dried in vacuum (40 °C, 24 h). The final product was obtained as a white powder with a yield of 43%.

The synthesis of octa-substituted carboxy-terminated POSS (OCPS) was carried out via acylation of OAPS with succinic acid [38]. In detail, to a solution of OAPS (15 g) and triethylamine (15 mL) in methanol (750 mL), succinic anhydride (60 g) was added, and the reaction mixture was stirred vigorously for 2 h at room temperature. White solid was afforded after precipitation in cold chloroform, filtration and washing with chloroform and THF. The crude product was further purified by being resolved in formic acid and reprecipitated in water, filtrated and washed with water until the pH value of the filtrate was 7. The solid was then dried in vacuum (40 °C, 24 h), to give a white powder with a yield of 69%.

### Synthesis of OCPS-intercalated Mg-Al LDH

The OCPS-intercalated Mg_2_Al_1_ LDH (denoted OLDH) was prepared via a one-step route [14]. Typically, in a 1 L flask equipped with a condenser, a pH meter, a nitrogen inlet tube and a magnetic stirrer, OCPS (8.40 g, 5.0 mmol) and water (200 mL) were placed under nitrogen. The mixture was stirred vigorously at room temperature and the pH value was adjusted to 10 by adding 1 M NaOH solution. After the system became clear and transparent, an aqueous solution containing Mg(NO_3_)_2_·6H_2_O (10.256 g, 40 mmol) and Al(NO_3_)_3_·9H_2_O (7.503 g, 20 mmol) in water (200 mL) was slowly added via micro syringe pump. During the synthesis, the pH value was maintained at 10 ± 0.02 by adding a suitable amount of NaOH solution. The resulting slurry was continuously stirred for 30 min and then allowed to age at 60 °C for more than 18 h. The precipitate was filtered and washed once with NaOH solution of pH 10, followed by thoroughly washing with water until the pH value of the filtrate was 7. The final product OLDH was obtained after drying at 80 °C in a blast drier for 4 h followed by 80 °C in vacuum for 24 h. In addition, unmodified pure LDH (NO_3_^−^-Mg-Al LDH, denoted NLDH) and sodium dodecylbenzenesulfonate-modified LDH (denoted DBS-LDH) were synthesized via the same method.

### Characterization

^1^H NMR and ^13^C NMR spectra were recorded in DMSO-*d*_6_ or D_2_O with a Bruker Avance III 400 spectrometer, and ^29^Si NMR spectra were recorded in DMSO-*d*_6_ with a Bruker DMX 500 spectrometer. FTIR spectra were measured with a Nicolet 560 spectrometer, using KBr pellets. X-ray diffraction was measured using an X’Pert PRO diffractometer with Cu Kα radiation (λ = 0.154 nm). Wide-angle X-ray scattering (WAXS) was measured in the range of 2θ = 3–70° with a step width of 0.026°, and small-angle X-ray scattering (SAXS) was measured in the range of 2θ = 1–10° with a step width of 0.013°. The LDH interlayer distance was calculated using Bragg’s law. Elemental analysis was performed using an Elementar Vario MICRO cube. Samples were analyzed in triplicate with a mass of 2 mg for each measurement. Matrix-assisted laser desorption/ionization-time of flight (MALDI-TOF) mass spectra were recorded with a Bruker Daltonics UltrafleXtreme using methanol as solvent and 2,5-dihydroxybenzoic acid as matrix. X-ray photoelectron spectroscopy (XPS) was carried out on a VG ESCALABMK II electron energy spectrometer using Mg Kα (1253.6 eV) as the X-ray excitation source. Transmission electron microscopy (TEM) images were recorded on an HT-7700 microscope with an acceleration voltage of 100.0 kV and bright-field illumination. The samples were dispersed in methanol/water mixed solvent, dropped onto carbon-coated copper grid and dried in a fume hood before characterization. Scanning electron microscopy (SEM) images were recorded on an SU-70 or Utral 55 to study the surface morphologies. The samples were placed on a conducting carbon cement holder and were then coated with a thin layer of platinum using a sputter coater. Thermogravimetric analysis (TGA) was performed on a TA-Q500 thermogravimeter at a heating rate of 10 K·min^−1^from room temperature to 800 °C under a nitrogen flow of 100 mL·min^−1^ or air atmosphere of 60 mL·min^−1^ O_2_ stream and 40 mL·min^−1^ N_2_ stream. Each sample was measured in an alumina crucible in triplicate, with a copy mass about 15 mg. The combustion behavior of the LDHs was measured using an FTT MCC-1 microscale combustion calorimeter, at a heating rate of 1 K·s^−1^ from 50 to 700 °C under a nitrogen flow of 100 cm^3^·min^−1^. The volatile, anaerobic thermal degradation products were mixed with a 20 cm^3^·min^−1^ gas stream containing 20% O_2_ and 80% N_2_ prior to being added to a 900 °C combustion furnace. Each sample was measured in triplicate with a copy mass of about 5mg, and the results were averaged.
